# C9orf72, a protein associated with amyotrophic lateral sclerosis (ALS) is a guanine nucleotide exchange factor

**DOI:** 10.7717/peerj.5815

**Published:** 2018-10-17

**Authors:** Shalini Iyer, Vasanta Subramanian, K. Ravi Acharya

**Affiliations:** Department of Biology and Biochemistry, University of Bath, Bath, UK

**Keywords:** C9orf72, Guanine nucleotide exchange factor, Protein expression, Protein purification, Size-exclusion chromatography, Rab GTPases, ALS, Neurodegeneration

## Abstract

Amyotrophic lateral sclerosis (ALS) and frontotemporal dementia (FTD), two late onset neurodegenerative diseases, have been shown to share overlapping cellular pathologies and genetic origins. Studies suggest that a hexanucleotide repeat expansion in the first intron of the C9orf72 gene is the most common cause of familial FTD and ALS pathology. The C9orf72 protein is predicted to be a differentially expressed in normal and neoplastic cells domain protein implying that C9orf72 functions as a guanine nucleotide exchange factor (GEF) to regulate specific Rab GTPases. Reported studies thus far point to a putative role for C9orf72 in lysosome biogenesis, vesicular trafficking, autophagy and mechanistic target of rapamycin complex1 (mTORC1) signaling. Here we report the expression, purification and biochemical characterization of C9orf72 protein. We conclusively show that C9orf72 is a GEF. The distinctive presence of both Rab- and Rho-GTPase GEF activities suggests that C9orf72 may function as a dual exchange factor coupling physiological functions such as cytoskeleton modulation and autophagy with endocytosis.

## Introduction

Frontotemporal dementia and amyotrophic lateral sclerosis (ALS) are two rapidly progressive debilitating neurodegenerative disorders. These two neurodegenerative diseases have long been linked both clinically as well as pathologically (Andersen & Al-Chalabi, 2011; Lomen-Hoerth, Anderson & Miller, 2002). One of the unifying pathogenic-signatures, mechanistically linking the two diseases, is a hexanucleotide repeat expansion (GGGGCC) in the first intron of the *C9orf72* gene (DeJesus-Hernandez et al., 2011; Renton et al., 2011). In wild type alleles there are, on average, three of these hexanucleotide-repeat units, with the maximum number increasing to only 30 units. However, in persons with the repeat expansion mutation there can be hundreds or even thousands of these repeat units (DeJesus-Hernandez et al., 2011; Renton et al., 2011). The inheritance of the *C9orf72* mutation is autosomal dominant, although reduced penetrance has been observed in some cases (Bigio, 2012; Van Blitterswijk, DeJesus-Hernandez & Rademakers, 2012). The repeat expansion has been shown to be unstable and repeat numbers have been shown to increase over generations (Vance et al., 2006).

Pathogenic repeat disorders, depending on their location, often cause either a loss or gain of function. Loss of function disease mechanism for C9orf72 arises when the expanded repeats in non-coding regions affect the expression levels of C9orf72 transcripts (DeJesus-Hernandez et al., 2011; Belzil et al., 2013; Donnelly et al., 2013). This results in a loss of function of the protein products alongside generating nuclear RNA foci (Wojciechowska & Krzyzosiak, 2011). The toxic gain of function, on the other hand, arises through accumulation in the frontal cortex and spinal cord of RNA transcripts containing the repeat (Van Blitterswijk et al., 2012), through the aberrant translation of dipeptide repeat species resulting from the C9orf72 repeat expansion (Ash et al., 2013; Mori et al., 2013), forming multiple RNA foci in both the cytoplasm and the nucleus (DeJesus-Hernandez et al., 2011).

Bioinformatics analysis has shown that there is secondary structure similarity between full-length C9orf72 and differentially expressed in normal and neoplastic cells (DENN)-like proteins (Levine et al., 2013; Zhang et al., 2012) DENN. DENN-domain containing proteins function as guanine nucleotide exchange factors (GEFs) and regulate GTPase-mediated membrane trafficking pathways. In cells, C9orf72 co-localizes with Rab proteins 7 and 11 suggesting that it is involved in the regulation of vesicular membrane traffic (Farg et al., 2014). C9orf72 is also implicated in the formation of autophagosome (Webster et al., 2016) and in the removal of aggregated proteins via the autophagy receptor, p62 (Sellier et al., 2016). Recently, C9orf72 has been shown to form a tri-molecular complex with SMCR8 (Amick, Roczniak-Ferguson & Ferguson, 2016) and WDR41 (Sullivan et al., 2016) upon depletion of amino acids in the cell. This complex regulates signal transduction via mechanistic target of rapamycin complex1.

Although C9orf72 is predicted to have GEF activity, there have been no reports so far demonstrating this. Herein, we describe the cloning, expression and purification of recombinant C9orf72 and address the choice of parameters that ensures optimal yield of the purified protein. We demonstrate that C9orf72 is a GEF for both Rab- and Rho-GTPases. We also show that C9orf72 is able to form a complex with each of the three Rab-GTPases studied. Finally, we also predict Rab-binding determinants on C9orf72 using computational methods.

## Materials and Methods

### Materials

*Strains.* High efficiency competent Stellar cells (*F−, endA1, supE44, thi-1, recA1, relA1, gyrA96, phoA*, Φ*80d lacZ*Δ *M15*, Δ (*lacZYA—argF) U169*, Δ *(mrr—hsdRMS—mcrBC)*, Δ*mcrA,* λ−) used for transformation of expression plasmids were ordered from Clontech (Takara Bio USA, Inc., Mountain View, CA, USA). Baculovirus DNA (bacmid) was a kind gift from Professor Ian Jones (University of Reading). Gibco^®^ Sf9 cells for insect cell expression and HEK293T/17 cells (ATCC^®^ CRL-11268™) for mammalian cell expression were sourced from ThermoFisher Scientific (Loughborough, UK) and American Type Culture Collection (ATCC; http://www.atcc.org/). Both insect and mammalian cell cultures were thawed/passaged/frozen/ according to the manufacturer’s protocol.

*Vectors.* Multiple host-enabled pOPIN series of vectors from Oxford Protein Production Facility (Didcot, UK) were used for the construction of expression plasmids to provide different fusion tags at the N- and/or C-terminal ends of C9orf72.

*Reagents.* High fidelity Polymerase Chain Reaction (PCR) system using KOD Hot Start DNA Polymerase (71086), GeneJuice^®^ (70967-3) and Luminata™ Forte western Horseradish Peroxidase (HRP) substrate (WBLUF0100) was obtained from Merck Millipore, USA. One kb DNA ladder (G5711), FuGENE^®^ HD (E2311), Wizard^®^ SV Gel and PCR Clean-Up System (A9282) and Wizard^®^ *Plus* SV Minipreps DNA Purification System (A1460) were purchased from Promega (Madison, WI, USA). Restriction enzymes used to linearise the bacmid DNA (*Bsu36I*) and pOPIN vectors (*KpnI*, *NcoI*) were purchased from New England Biolabs (Hitchin, UK). Agarose (A9539), sodium chloride (31434), HRP-conjugated polyclonal mouse (secondary) antibody, raised in goat (A4416) were obtained from Sigma-Aldrich (St Louis, MO, USA). Glycerol (10795711) was purchased Fisher Scientific (Hampton, NH, USA). Trypan blue/countess chamber slides (C10314), imidazole (10522714), β-mercaptoethanol (10367100) and Oxoid™ PBS tablets (BR0014G) were bought from ThermoFisher Scientific (Waltham, MA, USA). In-Fusion cloning kit (638916), monoclonal mouse anti-His (primary) antibody (MAB050) and HisTrap HP (five ml) column for affinity chromatography were sourced from R&D Systems (Minneapolis, MN, USA), Clontech (Takara Bio USA, Inc., Mountain View, CA, USA) and GE Healthcare (Buckinghamshire, UK), respectively.

*Growth medium.* Tryptone (T1332) and yeast extract (15159323) for bacterial cultures were purchased from Melford (Ipswich, Suffolk, UK) and Fisher Scientific (Hampton, NH, USA). Sf-900™ II Serum-Free Media (SFM) (10902096), Dulbecco’s Modified Eagle Media (DMEM) (41965039), trypsin-Ethylenediaminetetraacetic acid (EDTA) (25300062), non-essential amino acids (11140035), l-glutamine (25030024) and heat-inactivated foetal bovine serum (FBS) (10500064) were purchased from ThermoFisher Scientific (Waltham, MA, USA).

*Plastic-ware for cell culture work.* T75 flasks (3376), Erlenmeyer flasks (125 ml; 431143, 250 ml; 431144, 500 ml; 431145, 1 l; 431147, 2 l; 431255), six-well plates (3516), 12-well plates (3513) were purchased from Corning Life Sciences (Corning, NY, USA). 24-well plates were bought from VWR (Radnor, PA, USA). 24-deep-well blocks (19583) and breathable, self-adhesive plate seals (E2796–3015) were purchased from Qiagen (Hilden, Germany) and Starlab UK (Milton Keynes, UK).

### Methods

#### Construction of expression plasmids for C9orf72

The coding sequence of C9orf72 was amplified from the IMAGE consortium cDNA clone using KOD Hot Start DNA Polymerase according to the manufacturer’s instructions and inserted into several different expression vectors. PCR primers were designed with appropriate extensions to enable In-Fusion cloning (Table 1). The PCR products were separated by electrophoresis on a 1.2% w/v Tris-acetate EDTA (TAE) agarose gel and extracted from the gel using Promega’s Wizard^®^ SV Gel and PCR Clean-Up System. PCR products, eluted in nuclease-free water, were assessed for their yield (A260 measurement) and purity (A280/260) ratio using the NanoDrop 1000 Spectrophotometer. The pOPIN vectors were linearised with appropriate restriction enzymes. Restriction enzyme digests were subjected to agarose gel electrophoresis prior to gel extraction. In-Fusion cloning reactions were carried out by mixing 200 ng of the purified PCR product with 100 ng of the appropriately linearised expression vector and incubating the reaction at 42 °C for 30 min. High efficiency competent cells, Stellar cells (50 μl), were transformed with 2.5 μl of the In-Fusion reaction mixture. The transformation reactions were plated on Lysogeny Broth (LB)/agar plates containing ampicillin (100 μg/ml), 0.02% X-gal (to allow for blue–white screening) and one mM Isopropyl β-D-1-thiogalactopyranoside (IPTG), followed by overnight incubation at 37 °C. Recombinant clones (three to four white colonies per construct) were analyzed for authenticity by means of PCR and DNA sequencing. Sequence verified expression plasmids to be used for transfections into sf9 and/or HEK293T cells were prepared under sterile conditions using Wizard^®^ *Plus* SV Minipreps DNA Purification System according to manufacturer’s instructions and assessed for their yield (A260 measurement) and purity (A280/260) ratio using the NanoDrop 1000 Spectrophotometer.

**Table 1:** Cloning details of constructs of C9orf72, Rab GTPases.

C9orf72 constructs with different tags				
##	Vector	Tag	Forward primer	Reverse primer
1	pOPIN-Eneo	C-terminal His_8_	aggagatataccatgTCGACTCTTTGCCCACCGCCATC	gtgatggtgatgtttAAAAGTCATTAGAACATCTCGTTCTTGCACACTAGTG
2	pOPIN-F	N-terminal His_6_	aagttctgtttcagggcccgTCGACTCTTTGCCCACCGCCATC	atggtctagaaagctttaAAAAGTCATTAGAACATCTCGTTCTTGCACACTAGTG
3	pOPIN-S3C	N-terminal His_6_-SUMO	aagttctgtttcagggcccgTCGACTCTTTGCCCACCGCCATC	atggtctagaaagctttaAAAAGTCATTAGAACATCTCGTTCTTGCACACTAGTG
4	pOPIN-TRX	N-terminal His_6_-TRX	aagttctgtttcagggcccgTCGACTCTTTGCCCACCGCCATC	atggtctagaaagctttaAAAAGTCATTAGAACATCTCGTTCTTGCACACTAGTG
5	pOPIN-J	N-terminal His_6_-GST	aagttctgtttcagggcccgTCGACTCTTTGCCCACCGCCATC	atggtctagaaagctttaAAAAGTCATTAGAACATCTCGTTCTTGCACACTAGTG
6	pOPIN-M	N-terminal His_6_-MBP	aagttctgtttcagggcccgTCGACTCTTTGCCCACCGCCATC	atggtctagaaagctttaAAAAGTCATTAGAACATCTCGTTCTTGCACACTAGTG
7	pOPIN-HALO7	N-terminal His_6_-HALO7	aagttctgtttcagggcccgTCGACTCTTTGCCCACCGCCATC	atggtctagaaagctttaAAAAGTCATTAGAACATCTCGTTCTTGCACACTAGTG
8	pOPIN-E-3C-HALO7	C-terminal 3C-HALO7	aggagatataccatgTCGACTCTTTGCCCACCGCCATC	cagaacttccagtttAAAAGTCATTAGAACATCTCGTTCTTGCACACTAGTG

#### Transient expression of C9orf72 constructs in HEK293T cells

DMEM supplemented with 10% FBS, 1× non-essential amino acids and 1 mM l-glutamine was used to maintain HEK293T cultures. Cells were passaged when about 90% confluent. Transfections were carried out in 24-well plates, whereby each well was seeded with cells at a density of 2 × 10^5^ cells/ml (one ml per well) and incubated overnight at 37 °C in a 5% CO_2_/95% air environment. Next day, when cells were about 70% confluent, media in each of the wells were carefully aspirated and discarded. One ml fresh media (DMEM containing 2% FBS) were added to each well. Transfection reactions were prepared in PCR plates. For each transfection, one μg of plasmid DNA was mixed with 60 μl of serum-free DMEM and two μl of GeneJuice (at 1.33 mg/ml). The reaction mixture was mixed thoroughly and incubated at room temperature for 30 min. After the incubation period, the transfection-complexes were added to the cells in appropriate wells. The transfection plate was then incubated for 3 days at 37 °C in a 5% CO_2_/95% air environment.

For expression analysis, media from the wells were discarded and the plate with the cells was frozen at −80 °C for at least 30 min. Cell lysis was initiated by defrosting the plate at room temperature for 10 min, followed by addition of lysis buffer (50 mM NaH_2_PO_4_, 300 mM NaCl, 1% Tween-20 and 10 mM Imidazole). The cells detached from the wells and left to incubate at room temperature for 30 min with shaking at 300 rpm. The cell lysate was transferred to Eppendorf tubes and centrifuged for 30 min at 6,000×*g*. The supernatant for each transfection was analyzed by SDS–PAGE followed by detection (western blot) using a monoclonal mouse anti-His (primary) antibody (1:5,000) in combination with an HRP-conjugated polyclonal mouse (secondary) antibody (1:10,000), raised in goat and Luminata™ Forte western HRP substrate.

#### Transfection and expression screening of C9orf72 constructs in Sf9 cells

Serum-free Sf-900™ II SFM medium was used to maintain Sf9 cultures. Cells were allowed to reach a minimum density of 2.0 × 10^6^ viable cells/ml before sub-culturing to maintain cells in mid-log growth. Transfections were carried out in 24-well plates, whereby each well was seeded with cells at a density of 5 × 10^5^ cells/ml (500 μl per well) and left to adhere to the wells for an hour at room temperature. For each transfection, 0.5 μg of plasmid DNA was mixed with 250 ng of linearised bacmid DNA in 50 μl of serum-free media. To this 1.5 μl of FuGENE HD was added and the reaction mixture mixed thoroughly and incubated at room temperature for 30 min. After the incubation period, the transfection-complexes were added to the cells slowly, without disrupting the monolayer, in appropriate wells. The transfection plate was then gently swirled to distribute the mix across the well uniformly. The transfection plate was incubated at 27 °C for 6–7 days inside a lunch box lined with wet paper towels (to achieve a humid environment). At the end of the incubation period, the primary viral stocks (P0 virus) for each construct was harvested and stored in 10% FBS at −80 °C. *Sf9* monolayer (at 1 × 10^6^ cells/ml in 24-well plate; 0.5 ml per well) were infected with five μl P0 virus to generate P1 virus stock (incubation and long-term storage of the P1 virus stocks as described for P0 virus stocks).

For expression screening, 24 deep well plates (with round bottoms) were prepared with three ml *Sf9* cultures at 1 × 10^6^ cells/ml. Three μl or 30 μl P1 virus was added to each well and incubated at 27 °C for 3 days with shaking at 250 rpm. At the end of the incubation period, one ml from each well was transferred to 1.5 ml Eppendorf tubes and samples centrifuged for 30 min at 6,000×*g*. Supernatant was discarded and the pelleted cells were lysed and analyzed by SDS–PAGE and western blotting (as described in the previous section).

#### Viral amplification (suspension infection) to generate scaled P2 virus stock

Viral amplification was carried out in 250 ml Erlenmeyer flasks by preparing 50 ml of *Sf9* culture at 1 × 10^6^ cells/ml. A total of 400 μl P1 virus was added to the cells and incubated 27 °C for 6–7 days with shaking at 250 rpm. At the end of the incubation period, the culture was transferred to sterile tubes and centrifuged for 10 min at 1,000×*g*. The supernatant were transferred to fresh sterile tubes and filter sterilized by passing through a 0.2 μm sterile filter. Four ml aliquots of the filter-sterilized P2 virus stock (supplemented with 10% FBS) were prepared in sterile black tubes and stored at −80 °C. The P2 virus stock was then used to optimize viral titre and length of expression. Expression screening was carried out (in 125 ml flasks) as described above using 1, 10 and 25 μl P2 virus stock per 25 ml *Sf9* culture at 1 × 10^6^ cells/ml. Time points investigated were 72 and 120 h post-infection. At the end of the incubation period, expression samples for each viral titre and time point investigated were analyzed by SDS–PAGE and western blotting (as described in the previous section).

#### Large-scale expression of C9orf72 protein

Two liter Erlenmeyer shake flasks were prepared with 0.5 l of *Sf9* culture at 1 × 10^6^ cells/ml. A total of 500 μl of P2 virus stock per 0.5 l of *Sf9* culture was added to the flasks and incubated at 27 °C for 3 days with shaking at 250 rpm. At the end of the incubation period, cells were harvested by centrifugation for 30 min at 6,000×*g*. Supernatant was discarded and the pellets flash frozen in liquid nitrogen. The pellets were stored at −80 °C.

#### Purification of C9orf72 by immobilized metal affinity chromatography

Cell lysis and protein extraction was performed on ice. All the buffers used for purification were always made fresh and autoclaved prior to use. Cells from ∼4 l of *Sf9* cultures weighing (approximately) 14 g was used per purification. Cells were dispersed in 42 ml phosphate-buffered saline (PBS) (30 ml buffer per 10 g of cells) supplemented with 5% glycerol. A high-pressure cell disruptor from Constant Systems Ltd was used to lyse the cells (30 K psi). The lysate was centrifuged at 30,000×*g* for 45 min at 4 °C. Following centrifugation, 10 μl of Benzonase was added to the clarified lysate and stirred for 20 min at 4 °C. The lysate was then passed through a 0.45 μm filter before being applied to a five ml HisTrap HP column (pre-equilibrated with lysis buffer) at a flow rate of 0.8 ml/min. The column was then washed with 10 column volumes of PBS. Recombinant C9orf72, captured on the column, was eluted using PBS supplemented with 300 mM imidazole (pH 7.4). The eluted protein was concentrated and further purified by size-exclusion chromatography using Superdex200 (GE Healthcare, Buckinghamshire, UK). The eluate absorbance was monitored at 280 nm and fractions corresponding to the major absorbance peak were pooled and concentrated.

#### Protein quantitation

Concentration of the purified proteins was determined from UV absorbance using the ε_280_ value as determined by Protparam tool. Total protein (milligram) and total target protein (milligram) was estimated using the Bradford method, using the Coomassie Plus reagent and analysis of the reference standard curve obtained for a series of bovine serum albumin (BSA) dilutions.

#### Circular dichroism spectroscopy

Circular dichroism (CD) spectra were collected for all the purified proteins (five μM) using the Chirascan CD spectrometer in a one mm path length cell at 20 °C. A total of 25 spectral accumulations were obtained at each wavelength, ranging from 200 to 280 nm, and averaged. The response time was 1 s with spectral bandwidth of 2.5 nm. Secondary structural elements were estimated using Contin-LL.

#### Cloning, expression and purification of Rab GTPases

The gene for each of the three Rab GTPase was amplified from their cDNA clone using KOD HS Polymerase (Novagen, Watford, UK). The PCR product was ligated into the cloning vector, pBlueScriptII KS(+), digested with *Sma*I restriction enzyme (Promega, Madison, WI, USA). Restriction digestion and DNA sequencing identified true recombinant clones. Rab GTPase fragments were excised from these pBlueScriptII KS(+) clones by digestion with *Bam*HI and *Xho*I and ligated with similarly digested pGEX-6p-1 vector (GE Healthcare, Buckinghamshire, UK) to generate the expression plasmids. BL21CodonPlus(DE3)RIPL cells were subsequently transformed with the expression plasmids for expression in *Escherichia coli*.

LB media (five ml supplemented with ampicillin and chloramphenicol at a final concentration of 100 and 34 μg/ml, respectively) was inoculated with glycerol stock stab of the desired expression strain and incubated overnight at 37 °C with shaking (200 rpm). Next day, one l of autoinduction media (supplemented with antibiotics as above) was inoculated with 0.1% of the overnight starter culture. Expression cultures were incubated at 25 °C for 24 h with shaking at (200 rpm). At the end of the 24 h incubation, cells were harvested by centrifugation and each one l pellet was divided into three parts. Pellets were stored at −80 °C prior to purification.

Cell lysis and protein extraction were carried out on ice. Cells obtained from 0.33 l bacterial culture were dispersed in 70 ml lysis buffer (20 mM Tris–HCl (pH 8.0), 150 mM NaCl, 5% glycerol and two mM Dithiothreitol (DTT)) and ruptured using the cell disruptor at 20,000 psi. Triton X-100, to a final concentration of 0.1% (v/v), was added to the lysate and gently stirred for a couple of minutes at 4 °C. The lysate was centrifuged for 30 min at 70,000×*g* at 4 °C. The supernatant was applied to a pre-equilibrated five ml GST Sepharose 4B column (GE Healthcare, Buckinghamshire, UK) at a flow rate of 0.2 ml/min. Column was washed with wash buffer (20 mM Tris–HCl, pH 8.0, 150 mM NaCl, two mM DTT) to remove unbound proteins following which bound GST-tagged Rab GTPase were eluted off the column in the same buffer supplemented with 10 mM reduced glutathione. Following elution, PreScission Protease was added to the GST-tagged Rab GTPase according to the manufacturer’s protocol. The protein cleavage mixture was extensively dialysed against the wash buffer (20 mM Tris–HCl, pH 8.0, 150 mM NaCl, two mM DTT) for 16 h at 4 °C to remove reduced glutathione from the sample. The cleavage mixture, post dialysis, was centrifuged to remove precipitated proteins. Clarified sample was applied to a pre-equilibrated five ml GST Sepharose 4B column (GE Healthcare, Buckinghamshire, UK) at a flow rate of 0.2 ml/min to remove the GST tag and the protease from the Rab GTPase (collected in the flow through). The flow through was applied to the column for a second time. Samples from every stage of purification were analyzed by SDS–PAGE to confirm the presence of pure proteins.

#### Far-western analysis

GTPases (bait proteins) to be investigated were diluted in protein dilution buffer (PBS plus 0.05% v/v Tween-20) and applied to dry nitrocellulose membrane, with at least one cm spacing between the centers of each dot. The membrane was allowed to dry completely before placing it in blocking buffer (5% w/v dried skimmed-milk solids in PBS plus 0.05% v/v Tween-20) for an hour (at 15 rpm using a roller rig) at room temperature. Membrane blocking was followed by incubation of the membrane with C9orf72 (prey protein) for at least 6–8 h at 4 °C with gentle agitation. After incubation with the prey protein, the membrane was washed with blocking buffer three times (5 min each) with gentle rotation/agitation. Once washed, the membrane was incubated overnight at 4 °C with polyclonal rabbit anti-C9orf72 antibody (1:1,000 in blocking buffer) with gentle rotation/agitation. Next day the primary antibody solution was removed and the membrane washed as before with blocking buffer. The membrane was then placed in HRP-conjugated secondary anti-rabbit IgG (raised in goat) solution (1:2,000) for 3 h with gentle rotation/agitation. Following incubation with the secondary antibody solution, the membrane was washed twice with blocking buffer (5 min each) and then twice more with protein dilution buffer for 5 min each. The blot was activated using Luminata™ Forte western HRP substrate and the signal detected using the Vilber FUSION-SL imaging system.

#### SEC binding studies

To study complex formation between the Rab proteins and C9orf72, 3× molar excess of the each Rab protein was mixed with C9orf72 and incubated overnight on ice in the presence of 20 mM EDTA. The protein mixture was injected onto a Superdex75 gel-filtration column (GE Healthcare, Buckinghamshire, UK) equilibrated with buffer containing 20 mM Tris (pH 8.0), 300 mM NaCl, two mM DTT and 20 mM EDTA. The sample was applied to the column using a 100 μl loop at 0.5 ml/min using an AKTA PrimePlus FPLC system (GE Healthcare, Buckinghamshire, UK). Elution profile was monitored by UV absorbance at 280 nm. These were compared to injections of Rab proteins alone. Fractions were analyzed by reducing analytical SDS–PAGE using Coomassie Blue staining and western blot.

#### Guanine nucleotide exchange factor assay

Guanine nucleotide exchange factor assays were performed using Promega’s GTPase-Glo™ Assay kit. C9orf72 and the GTPases were serially diluted in GEF buffer containing 10 μM guanosine-5′-triphosphate (GTP) and 6.25 μl of each dilution (in triplicate) was dispensed into the wells of a white half-area 96-well plate. The GTPase reaction was initiated by adding an equal volume of a GTPase in each well. The reaction was incubated at 22 °C for 120 min. Once the GTPase reaction was completed, 12.5 μl of appropriately diluted GTP-Glo™ reagent, at room temperature, was added to all the wells. The plate was incubated at 22 °C for 30 min, with gentle shaking. At the end of the incubation period, 25 μl of the Detection Reagent (provided with the kit) was added to all the wells. Endpoint luminescence signal was measured using a BMG Labtech microplate reader with a 1 s read time, after the plates were further incubated for 5–10 min at 22 °C.

#### Structure prediction and model validation

SOPMA (Geourjon & Deléage, 1995) analysis, PSIPRED (Buchan et al., 2013), RaptorX (Källberg et al., 2012) and LOMETS (Wu & Zhang, 2007) were used with default parameters to predict protein secondary structure. TM_align (Zhang & Skolnick, 2005) and COFACTOR (Zhang, Freddolino & Zhang, 2017) were used for validating the reliability of the models generated. Simple molecular dynamics followed by energy minimization of the homology model of C9orf72 generated by RaptorX was carried out using PHENIX (Adams et al., 2010) to ensure ideal bond length, bond angle and dihedral angles for all the residues. The energy minimized C9orf72 homology model was validated using ProSA (Wiederstein & Sippl, 2007), VERIFY_3D (Lüthy, Bowie & Eisenberg, 1992) and PROCHECK (Laskowski et al., 1993). ProSA (Wiederstein & Sippl, 2007) and VERIFY_3D (Lüthy, Bowie & Eisenberg, 1992) servers were used to assess energy profile plots of the model as an indicator of model quality, both overall and at the amino acid level. The overall stereo-chemical quality of the model was evaluated by calculating Ramachandran plots using PROCHECK (Laskowski et al., 1993). PROCHECK evaluates the model by calculating φ (phi) and Ψ (psi) angles of the amino acids in Ramachandran plot (Ramachandran, Ramakrishnan & Sasisekharan, 1963). VERIFY_3D (Lüthy, Bowie & Eisenberg, 1992) checks the quality of the model in terms of stereo-chemical properties of the amino acids and energetics by performing 1D sequence profiling, wherein the protein model is compared against its own sequence with the surrounding environment of atoms from the same protein. ProSA (Wiederstein & Sippl, 2007) produces an overall model quality index called the *z*-score based on statistical analysis of the total energy of the model with respect to the energy distribution of different conformations of amino acids derived from all the available structures in the Protein Data Bank (PDB) with the same number of amino acids. The energy profile plot, on the other hand, gives a measure of model quality at the amino acid level.

The idealized model was used to generate in silico complexes of C9orf72 with Rabs 5, 7 and 11. The homology model of C9orf72 was superposed onto the DENND1B component of the DENND1B-Rab35 complex (PDB code: 3TW8; Wu et al., 2011). Crystal structures of Rab5A (PDB code: 1N6H; Zhu et al., 2003), Rab7A (PDB code: 3LAW; McCray, Skordalakes & Taylor, 2010), Rab8a (PDB code: 4LHW; Guo et al., 2013) and Rab11A (PDB code: 1OIX; Pasqualato et al., 2004) were superposed on the GTPase component of the DENND1B-Rab35 complex (PDB code: 3TW8; Wu et al., 2011). Models of the three complexes were also put through simple molecular dynamics followed by energy minimization using PHENIX (Adams et al., 2010) to ensure ideal bond length, bond angle and dihedral angles for all the residues. These complexes were then analyzed for intermolecular contacts between the C9orf72 and Rab proteins using the program CONTACT (Winn et al., 2011).

## Results

### C9orf72 expression

cDNA for human C9orf72 was obtained from Source Bioscience (Clone id—IRATp970C0789D). The C9orf72 gene was initially cloned into a variety of vectors such as pTriEx-4 Ek/LIC (N-terminal His_6_ Tag), pET-SUMO (N-terminal SUMO-His_6_ tag), pGEX-6p-1 (N-terminal glutathione S-transferase (GST) tag), as well as a modified version of pMAL-p2X (N-terminal His_6_-MBP tag). Although both the GST-tagged and His_6_-MBP-tagged C9orf72 expressed in soluble form, the protein was unstable. The protein showed signs of degradation almost immediately post-lysis. Removal of the N-terminal tag (GST or the His_6_-MBP) always resulted in the protein precipitating out. We did not have much success with our constructs with smaller tags either. The SUMO-His_6_ tagged C9orf72 expressed as inclusion bodies and the construct with the minimal tag (His_6_) did not express at all in *E. coli*. A selection of bacterial expression strains was tried (such as Shuffle T7 express and Nico21(DE3) (from New England Biolabs, Hitchin, UK), Rosetta strains, Rosettagami strains (from Novagen, Watford, UK), SoluBL21 (from Amsbio, Abingdon, UK)), along with a variety of media types and expression trials varying different parameters, but none managed to improve expression levels or produced protein that was stable. We also prepared constructs where maltose-binding protein (MBP) and GST tags were moved to the C-terminal end of the protein but obtained similar results as above.

Producing a functional mammalian protein often necessitates the use of a eukaryotic expression host to ensure appropriate posttranslational modifications (e.g., disulphide bonds, glycosylation). Amino acid sequence analysis suggested that the C9orf72 protein might have a potential phosphorylation site as well as a glycosylation site. Bearing this in mind, we cloned the *C9orf72* gene into eight different vectors from the pOPIN suite of vectors (Table 1, provided by OPPF-UK) and carried out expression screening in both *Sf9* and HEK293T cells. The intention was to compare expression of the various constructs of C9orf72 in both insect and mammalian system and choose the vector/system that gave optimum expression.

**Figure 1:**
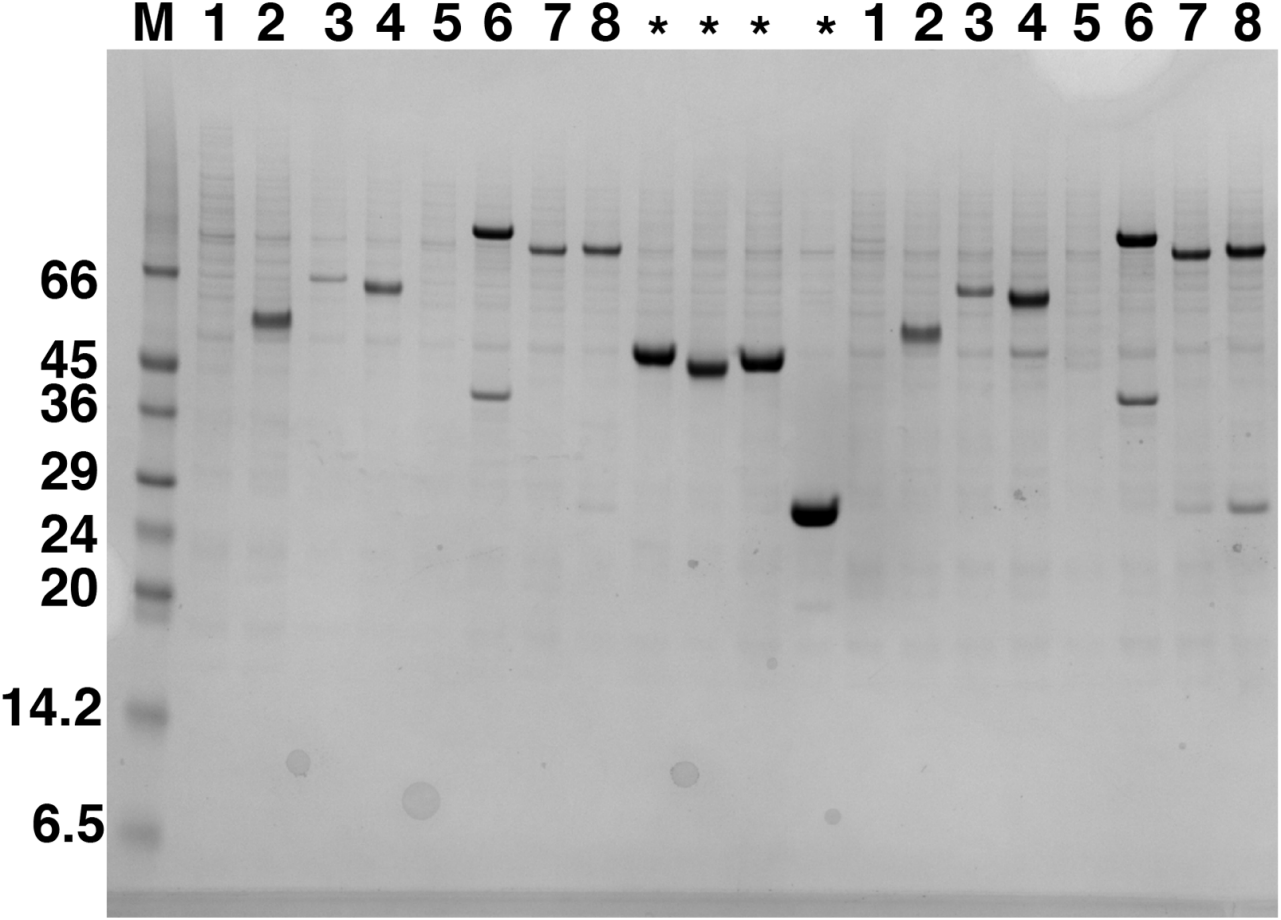
Western blot analysis of expression screening of C9orf72 in *Sf9* cells. Different constructs of C9orf72 (Table 1) were screened for expression in Sf9 cells. Expression was analyzed by western blotting as described in the materials and methods section. The lane labeled M represents the Low Range Sigma Markers (M3913). Molecular marker masses are indicated in kilodalton. Lanes are numbered 1–8, according to the construct numbers detailed in Table 1. Lane 2 showing expression for pOPINF-C9orf72 was selected for scale-up expression and purification. The lanes marked with asterisks are control proteins for which transfections were carried out alongside the eight C9orf72 constructs.

Expression screening of PCR verified clones in *Sf9* cells was followed by small-scale purification of soluble proteins using Nickel Nitrilotriacetic acid (Ni-NTA) magnetic beads and analyzed by SDS–PAGE. Expression from the different C9orf72 constructs, following transient transfection in HEK293T cells, was detected by western blotting. The results showed that only three of the eight constructs expressed C9orf72 protein in the soluble fraction in HEK293T cells. Expression of C9orf72 in pOPINS3C (N-terminal His_6_-SUMO tag) and pOPINM (N-terminal His_6_-MBP tag) gave the strongest signals in the western blot (for HEK293T cells), whilst pOPINTRX-C9orf72 (N-terminal His_6_-Thioredoxin tag) showed very low levels of expression. In contrast, expression in insect cells was more successful as six out of the eight constructs produced soluble C9orf72 (Fig. 1), demonstrating that *Sf9* system was the most suitable for C9orf72 expression. No expression was detected in the soluble fraction from vectors pOPINE (C-terminal His_6_ tag) and pOPINJ (N-terminal His_6_-GST tag). All the eight constructs of C9orf72 were sequence verified to ensure the gene was in the correct reading frame. There was not much difference in expression when comparing results from 3 and 30 μl P1/P2 virus infections. All subsequent experiments were performed using the minimally tagged (N-terminal His_6_) C9orf72 (pOPINF-C9orf72; Fig. 1) because it provided reasonable yields of the tagged protein, the tag added only ∼2 kDa to the mass of the protein and most importantly the protein could be purified from inexpensive, high capacity resin.

### C9orf72 expression optimization

**Figure 2:**
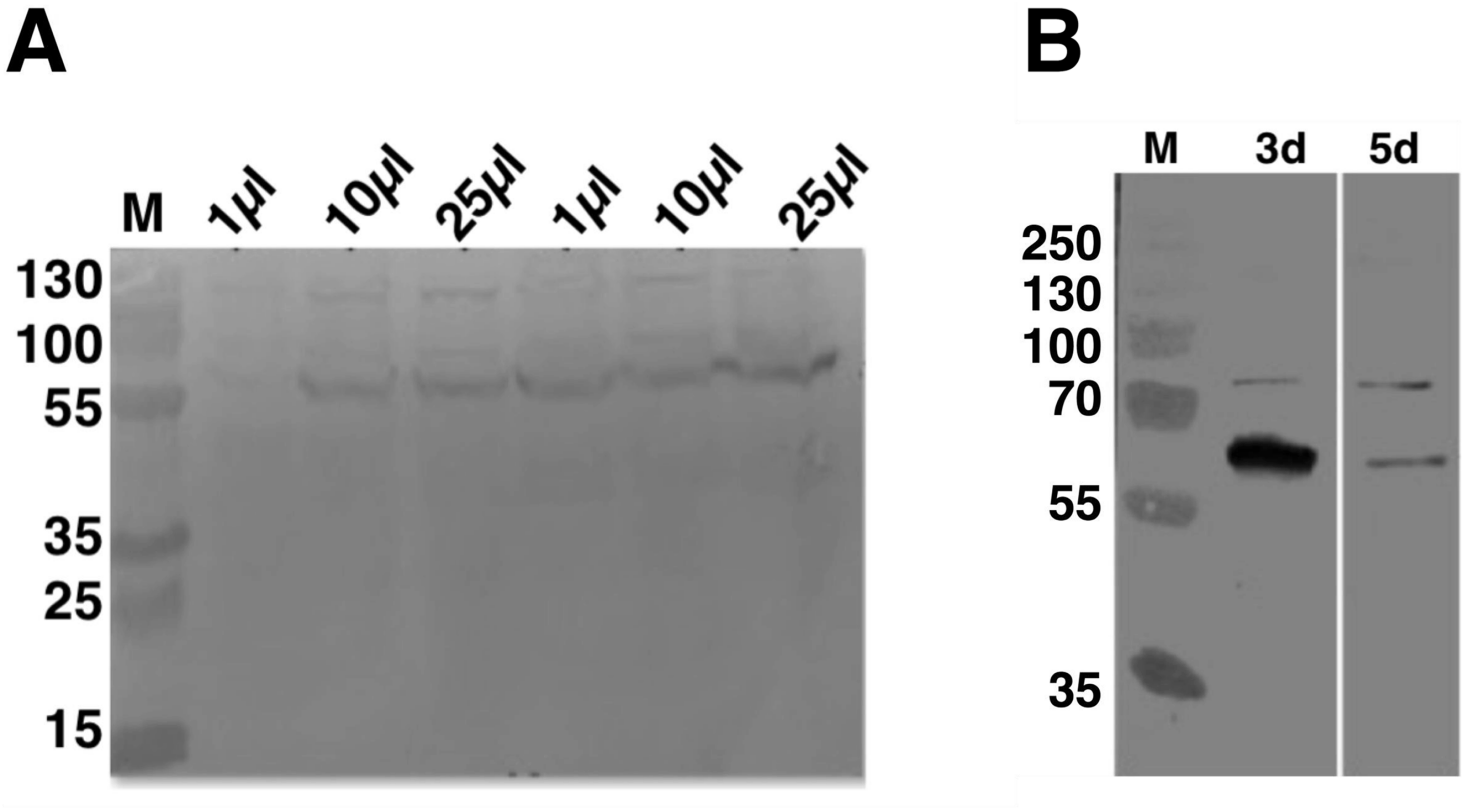
Analysis of expression optimization of C9orf72 in *Sf9* cells. pOPINF-C9orf72 construct selected for expression in *Sf9* cells was tested for (A) total volume of virus used by SDS–PAGE analysis. The two series of volumes (1, 10 and 25 μl) shown represent volumes of viruses generated from using two different concentrations of DNA during transfection. (B) Total time of infection (3d, 3 days and 5d, 5 days). Expression was analyzed by western blotting as described in the materials and methods section. Detection was carried out using a monoclonal mouse anti-His (primary) antibody (1:5,000) from R&D Systems (MAB050) in combination with an HRP-conjugated polyclonal mouse (secondary) antibody (1:10,000), raised in goat and Luminata™ Forte western HRP substrate. The lanes labeled M in both panels contain the PageRuler™ Plus Prestained Protein ladder (Fermentas). Molecular marker masses are indicated in kilodalton.

To establish the optimal volume of virus used and total time of infection for C9orf72 expression from *Sf9* cells, 25 ml insect cell cultures at 1 × 10^6^ cells/ml were infected with 1, 10 and 25 μl P2 virus stock (Fig. 2A). Aliquots of the infected cultures were taken 72 h post-infection for each viral titre tested. Analysis by means of western blotting revealed that there was a small difference in the expression levels for the 10 and 25 μl virus infections, with the latter showing slightly higher level of expression (Fig. 2A). The culture infected with only one μl of the virus produced only very small amount of protein (Fig. 2A). Following this, the culture that was infected with 25 μl virus was further incubated for another 2 days (total of 120 h post-infection). The results showed that prolonged expression led to marked reduction in C9orf72 expression levels (Fig. 2B). This indicated that longer incubation was not suitable possibly due to cell lysis and/or cell death as well as degradation of C9orf72. Hence, expression was routinely carried out by infecting *Sf9* cultures with 0.1% (v/v) of the amplified viral stock for 3 days at 27 °C with shaking at 140 rpm.

### Purification of His_6_-C9orf72 protein

**Figure 3:**
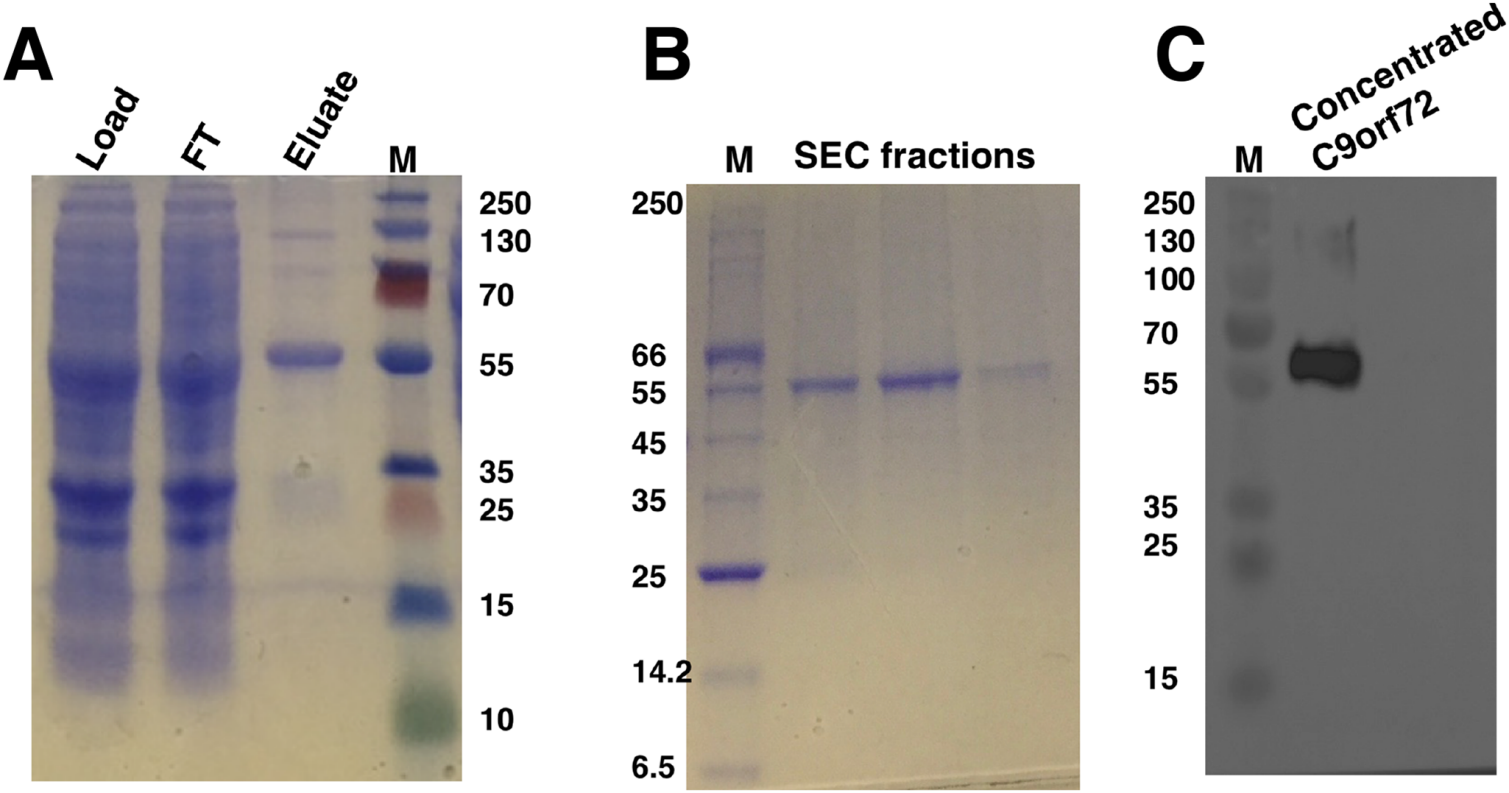
SDS–PAGE and western blot analysis of C9orf72 purification. (A) SDS–PAGE analysis of samples from Ni^2+^-affinity purification of C9orf72 (B) SDS–PAGE analysis of fractions from size-exclusion chromatography purification of C9orf72 and (C) western blot analysis of concentrated C9orf72. Detection was carried out using an affinity purified rabbit polyclonal anti-C9orf72 (primary) antibody (1:1,000) from Santa Cruz Biotechnology (sc-138763) in combination with an HRP-conjugated polyclonal rabbit (secondary) antibody (1:10,000), raised in goat and Luminata™ Forte western HRP substrate. The lanes labeled M in all three panels contain the PageRuler™ Plus Prestained Protein ladder (Fermentas). Molecular marker masses are indicated in kilodalton.

The optimal conditions identified for expression were adopted for expressing C9orf72 in larger Sf9 cultures (14 g cell pellet per purification was used). Preliminary investigations revealed that temperature played an important role in the stability of C9orf72. Keeping the protein on ice throughout the purification process and addition of glycerol alleviated the problem of protein aggregation as well as degradation to a large extent. Recombinant C9orf72 could be captured in a two-step purification process involving Ni^2+^ affinity (Fig. 3A) and size-exclusion chromatography (Fig. 3B), authenticity of which was confirmed by western blotting (Fig. 3C). Both anti-C9orf72 antibody as well as the anti-His antibody recognized the purified protein as C9orf72. The presence of dimers in the purified protein and sometimes even higher oligomers, as revealed by the western blots, showed the propensity of C9orf72 to form SDS-resistant aggregates/oligomers. Protein yield was between 0.8 and 1.0 mg/ml from one l of insect cell culture. Purity of C9orf72 was assessed by SDS–PAGE analysis and western blot. Purity was judged to be >95% by these techniques.

**Figure 4:**
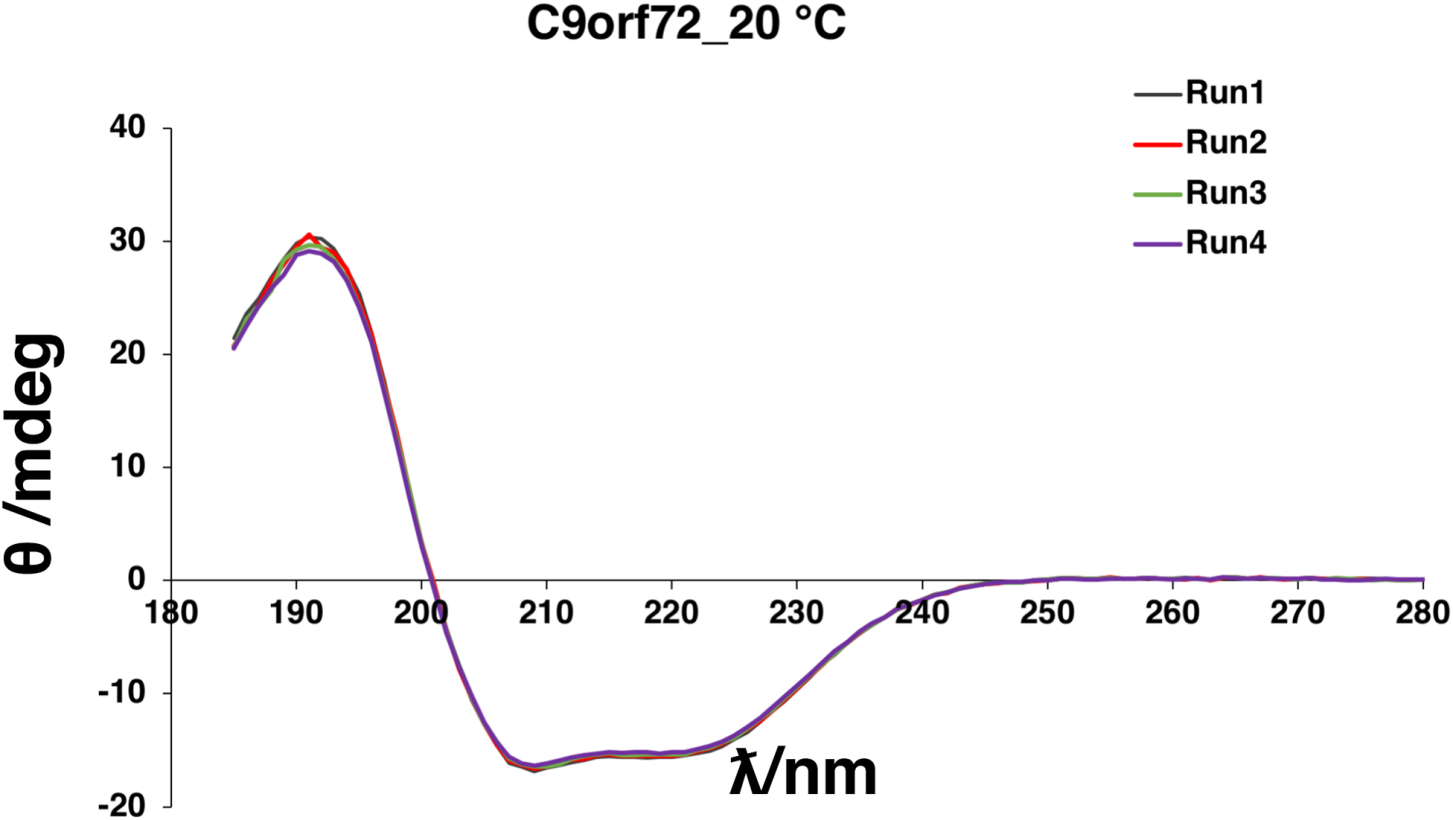
Far-UV CD spectrum showing native state of C9orf72. CD spectra were collected for purified C9orf72 (five μM) using the Chirascan CD spectrometer in a one mm path length cell at 20 °C between 185 and 280 nm. The response time was 1 s with spectral bandwidth of 2.5 nm. Secondary structural elements were estimated using the K2D algorithm (Andrade et al., 1993) included with DichroWeb (Whitmore & Wallace, 2007).

Circular dichroism spectroscopy was used to validate the folded state of purified C9orf72 and to distinguish secondary structure content of the protein (Fig. 4). The overall shape of the far-UV spectrum observed for C9orf72 characterizes the protein to be predominantly α-helical. Looking at the spectral features of C9orf72, we can see a double minimum at 208 and 221 nm, features that are characteristic of α-helical proteins like myoglobin or poly-l-lysine (commonly used as a reference standard in CD spectroscopy). This observation was further validated by software packages such as Contin-LL, K2d and Selcon 3 (all three programs are supported by DichroWeb). These programs also predicted C9orf72 to be predominantly α-helical in nature.

### Expression and purification of Rab GTPases

**Figure 5:**
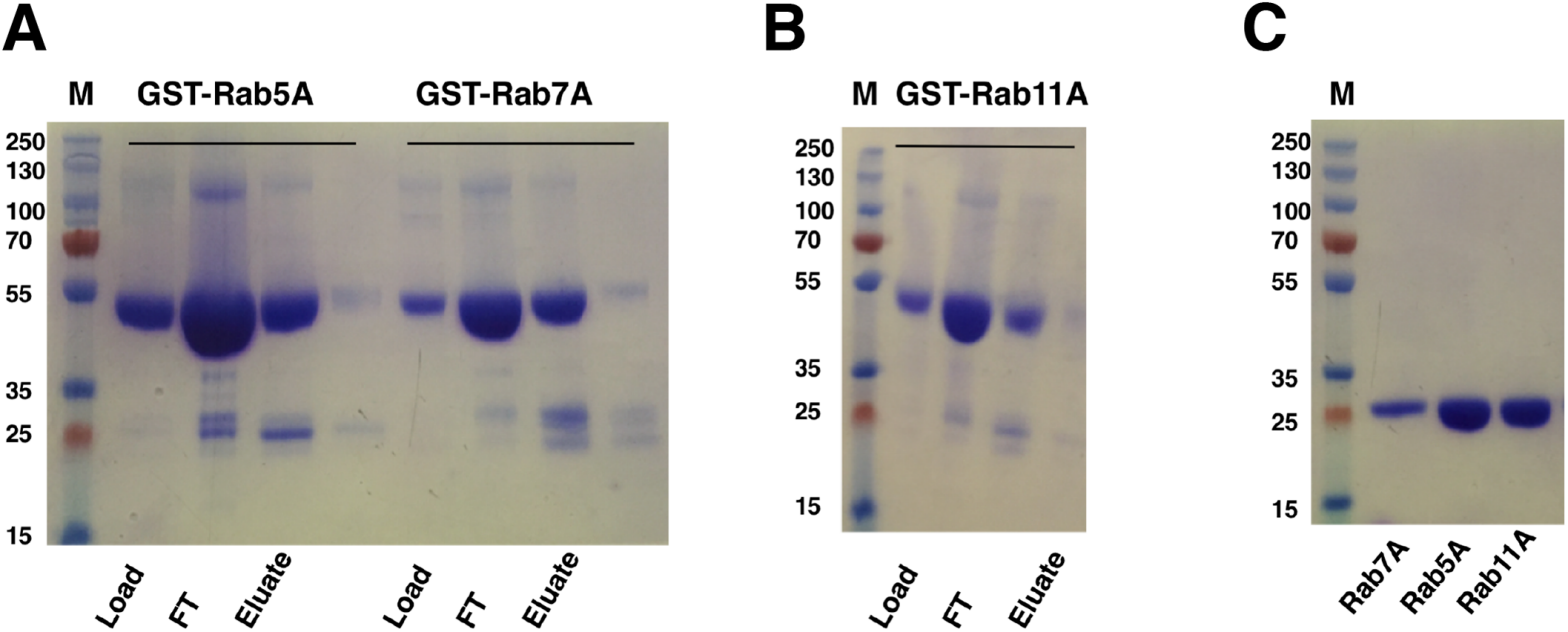
SDS–PAGE analysis of Rab GTPase purifications. (A and B) SDS–PAGE analysis of eluted samples from GST-affinity purification of Rab GTPases, GST-Rab5A, GST-Rab11A and GST-Rab7A. (C) SDS–PAGE analysis of Rab GTPases after tag cleavage. Molecular marker masses, indicated in kilodalton, were determined by the PageRuler™ Plus Prestained Protein ladder (Fermentas). Molecular mass of GST-tagged Rab GTPases is around 50 kDa. After tag cleavage the Rab GTPases have a molecular mass of around 25 kDa.

*Escherichia coli* cells harboring the Rab gene (Rab5A, Rab7A or Rab11A) in pGEX-6p-1 vector expressed the GST-tagged Rab proteins at around 50 kDa, corresponding to the predicted mass of the fusion proteins. GST-Rab fusion proteins were purified from the supernatants of cell lysates using a five ml GST Sepharose 4B column (GE Healthcare, Buckinghamshire, UK). Elution with reduced glutathione buffer yielded good amounts of the fusion proteins (Figs. 5A and 5B). Cleavage of GST-tag from the Rab proteins was achieved by combining the cleavage reaction with overnight dialysis in a buffer devoid of reduced glutathione. This ensured that the cleavage reaction could be applied onto a washed and pre-equilibrated GST-affinity column to separate the GST-tag from the Rab proteins. Since PreScission Protease (GE Healthcare, Buckinghamshire, UK) is also GST-tagged, reapplication of the cleavage reaction to the GST Sepharose 4B column (GE Healthcare, Buckinghamshire, UK) also helped in separating the protease from the protein of interest. The tag-cleaved Rab proteins were collected in the flow-through. Applying the flow-through to the column for a second time helped in removing residual GST from the protein sample. About 17 mg of Rab5A, six mg of Rab7A and 18 mg of Rab11A (from a 333 ml starting culture) with almost 100% purity could be achieved by this method (Fig. 5C).

### Protein–protein interaction

**Figure 6:**
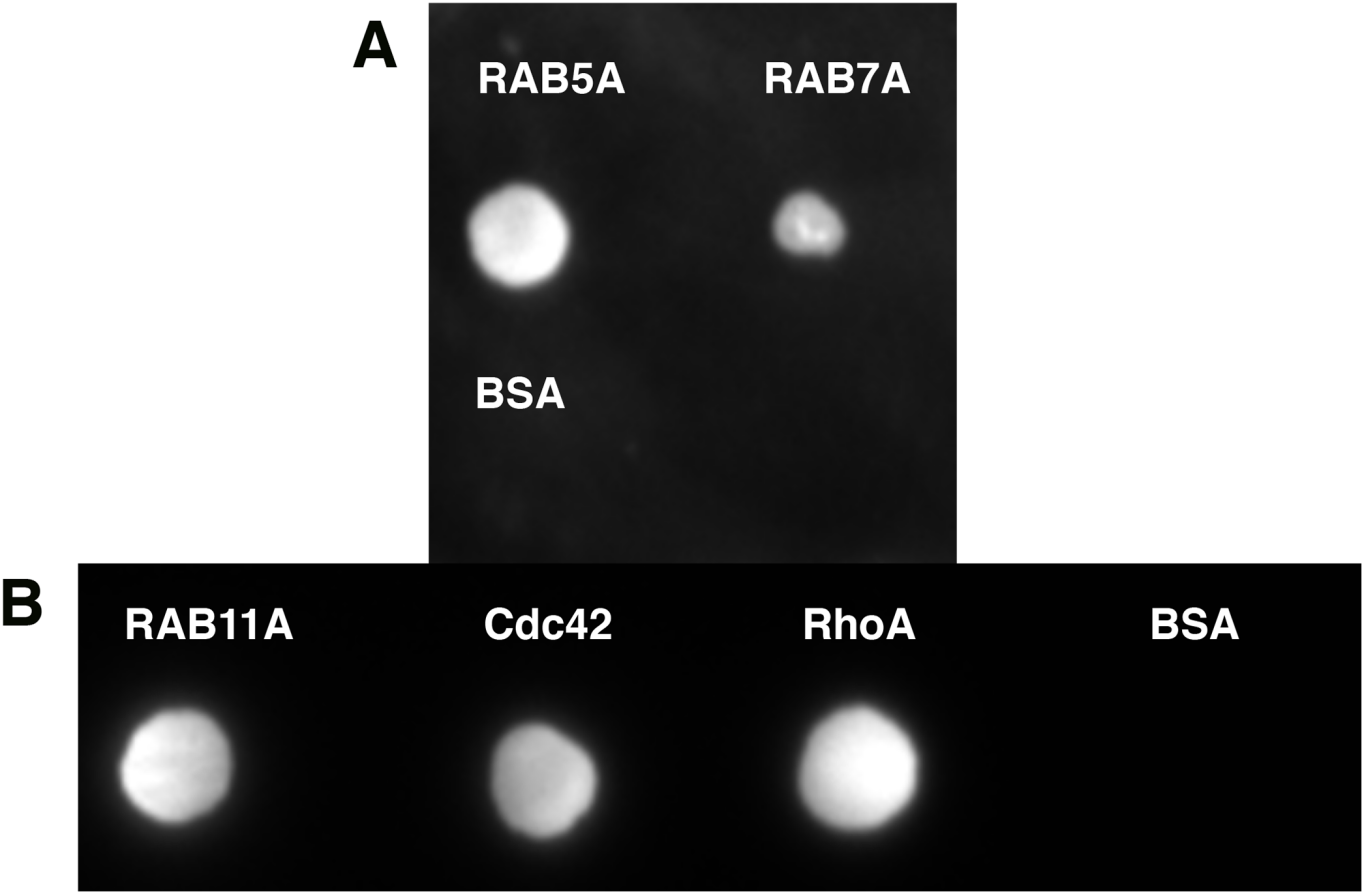
Far-western analysis to assess protein–protein interactions. (A) Rab5A, Rab7A and BSA were spotted at 50 pmoles on a nitrocellulose membrane. BSA was included as a negative control. (B) Rab11A, Cdc42 and RhoA were spotted at 50 pmoles on a nitrocellulose membrane. BSA was included as a negative control. The membrane was incubated with 100 nM purified C9orf72 (prey protein) for at least 6–8 h at 4 °C with gentle agitation. This was followed by incubation with the primary antibody, polyclonal rabbit anti-C9orf72 antibody (1:1,000 in blocking buffer). The membrane was subsequently incubated with the HRP-conjugated secondary goat anti-rabbit antibody at a 1:2,000 dilution.

**Figure 7:**
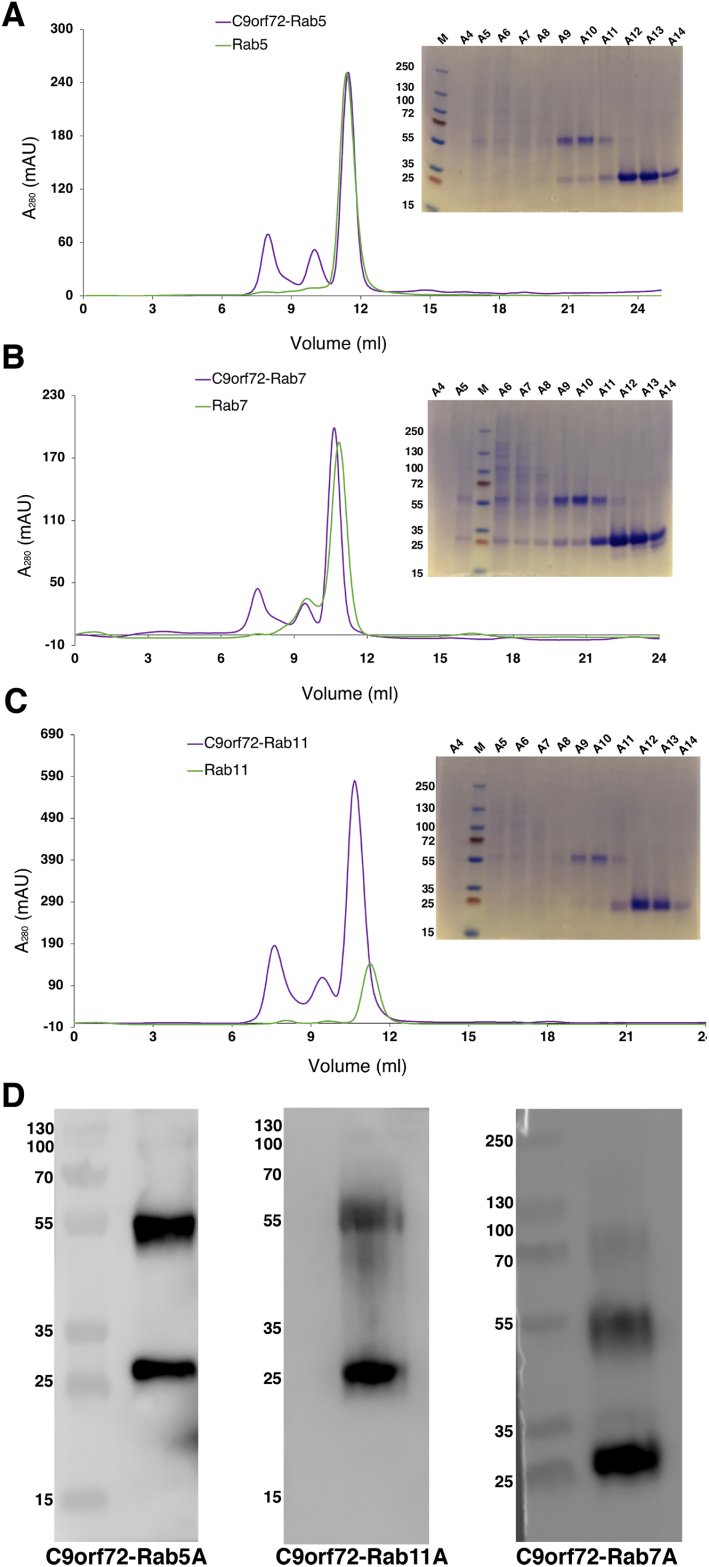
Complex formation of the Rab proteins with C9orf72, analyzed by size-exclusion chromatography. Complex formation of Rab5A (A), Rab7A (B) and Rab11A (C) with full-length C9orf72 was analyzed on a Superdex 200 10/30 column (GE Healthcare, Buckinghamshire, UK) by monitoring the UV absorption at 280 nm (left). Eluted fractions were subjected to denaturing polyacrylamide gel electrophoresis (SDS–PAGE) to verify Rab and C9orf72 co-elution (right). An amount of 21 μM C9orf72 and 48 μM Rab were applied per gel filtration run. (D) Western blot analysis of the eluted complexes: C9orf72-Rab5A (left panel), C9orf72-Rab11A (middle panel) and C9orf72-Rab7A (right panel). The band around the 55 kDa mark is C9orf72 and the band at around 25 kDa mark all the three panels is the Rab protein from the respective complex. Detection was carried out using an affinity purified rabbit polyclonal anti-C9orf72 (primary) antibody (1:1,000) from Santa Cruz Biotechnology (sc-138763) in combination with: anti-Rab5A (primary) antibody (1:400) for C9orf72-Rab5A complex, anti-Rab7A (primary) antibody (1:200) for C9orf72-Rab7A complex and anti-Rab11A (primary) antibody (1:200) for C9orf72-Rab11A complex. The Rab antibodies were from a sampler kit bought from Cell Signaling Technology (9385).

Quite often protein–protein interactions are essential for associated function of many biological systems. In the present case, far-western analysis was used as a rapid qualitative method to probe the previously hypothesized interaction between C9orf72 and Rab proteins (Farg et al., 2014). Purified Rab-GTPases (Rabs 5, 7 and 11) were immobilized on nitrocellulose membrane for a dot blot assay and then probed with C9orf72 protein. After washing, the binding interactions were detected by anti-C9orf72 antibody (Fig. 6). The experiment showed that all the three Rab proteins tested were able to bind C9orf72 as predicted by Farg et al. (2014). These findings prompted us to confirm this binding using analytical size-exclusion chromatography (Fig. 7). The eluted fractions were analyzed by SDS–PAGE (Figs. 7A, 7B and 7C) and western blot analysis (Fig. 7D). Mixing a 3×-molar excess of the Rab protein with C9orf72 in the presence of 20 mM EDTA led to the formation of a complex. All three Rab proteins eluted as a complex with C9orf72. Rab5A and Rab11A eluted as a 1:1 complex with C9orf72. The elution profile of Rab7A with C9orf72, on the other hand, could be interpreted as equilibrium between a 2:2 complex and a 2:1 complex (Fig. 7B).

### GEF activity of C9orf72

**Figure 8:**
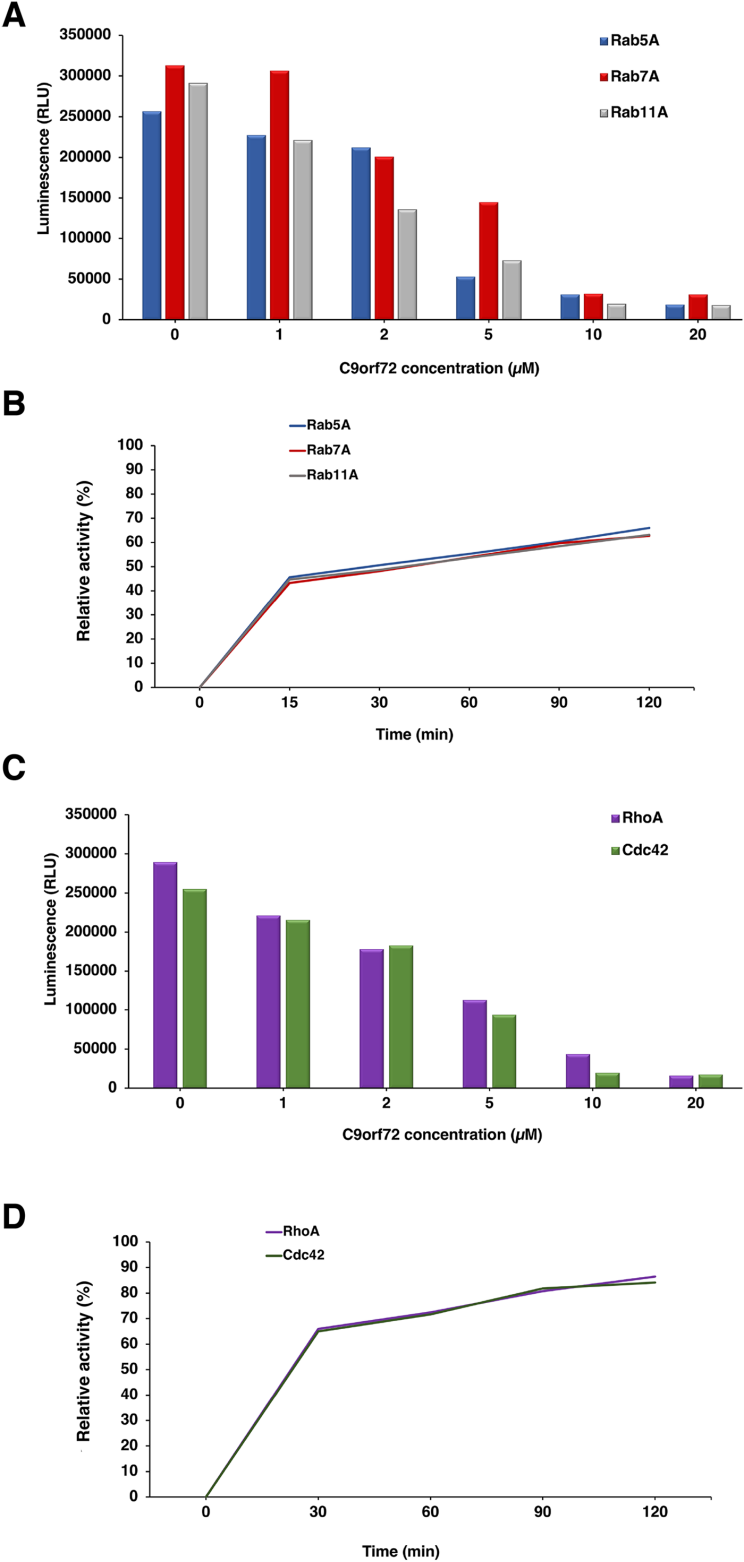
Guanine nucleotide exchange activity of C9orf72. (A) Titration of C9orf72 in the presence of fixed concentrations of Rab-GTPases. The reactions contained two μM of Rab-GTPase (Rab5A, Rab7A or Rab11A), one mM DTT, five μM GTP in GEF buffer along with different concentrations of C9orf72 (0–20 μM). The reactions (in triplicate) were incubated at 22 °C for 120 min. (B) Time course assay to determine optimal Rab-GTPase reaction time for C9orf72 at fixed concentration (determined above). The reactions contained two μM of Rab-GTPase (Rab5A, Rab7A or Rab11A), one mM DTT, five μM GTP in GEF buffer along five μM C9orf72. The reactions (in triplicate) were incubated at 22 °C for different time periods. (C) Titration of C9orf72 in the presence of fixed concentrations of Rho-GTPases. The reactions contained two μM of Rho-GTPase (Cdc42 or RhoA), one mM DTT, five μM GTP in GEF buffer along with different concentrations of C9orf72 (0–20 μM). The reactions (in triplicate) were incubated at 22 °C for 120 min. (D) Time course assay to determine optimal Rho-GTPase reaction time for C9orf72 at fixed concentration (determined above). The reactions contained two μM of Rho-GTPase (Cdc42 or RhoA), one mM DTT, five μM GTP in GEF buffer along five μM C9orf72. The reactions (in triplicate) were incubated at 22 °C for different time periods.

The GEF activity of C9orf72 protein (Fig. 8) was determined using the GTPase Glo assay from Promega. We tested the GEF activity of C9orf72 by titrating C9orf72 in the presence of fixed concentration of Rab GTPases (Fig. 8A). We observed increasing GTP hydrolysis concomitant with lower luminescence signal when increasing the amount of C9orf72 used in the GTPase reaction (Fig. 8A). C9orf72 was found to be active on all the three Rab GTPases (Rab5A, Rab7A and Rab11A) tested. At the lowest concentration, Rab7A reaction was the slowest with only 2.1% of the reaction completed when compared to the other two Rab proteins. Activity of C9orf72 on Rab7A was the slowest for each of the five concentrations tested.

To study the appropriate incubation time for GTPase reactions, we also performed a time course experiment (Fig. 8B) whereby a fixed amount of Rab-GTPase was incubated with a fixed amount of C9orf72 for different time periods (0, 15, 30, 60, 90 and 120 min). As expected, longer incubation times allowed for more GTP hydrolysis. Around 45% of the reaction was complete in the first 15 min. Following which the reaction appeared to slow down. GTP hydrolysis reached only about 60–70% completion even after 2 h of incubation.

### C9orf72 and Rho-GTPases

In our far-western analysis to probe interactions between C9orf72 and the Rab-GTPases, we used the GTPases Cdc42 and RhoA as negative controls (purchased from Universal Biologicals, Cambridge, UK) in addition to BSA. Surprisingly, both these Rho-GTPases showed binding to C9orf72 (Fig. 8C). This prompted us to check if C9orf72 was able to mediate the exchange of Guanosine diphosphate (GDP) bound to these Rho-GTPases with GTP, thereby activating them. Interestingly, we observed significant GTP hydrolysis resulting in low luminescence signal in the GEF assay. The higher the concentration of C9orf72 lower was the light output. Similarly, to the GEF activity of C9orf72 on the three Rab-GTPases we tested, about 50% of the reaction is complete in the first 30 min after which the reaction slowed down (Fig. 8D).

This is the first report demonstrating the potential ability of C9orf72 to bind and catalyze guanine nucleotide exchange on members of the GTPase superfamily other than the Rab GTPases.

### Homology model of C9orf72 and its complex with Rab proteins 5, 7 and 11

**Figure 9:**
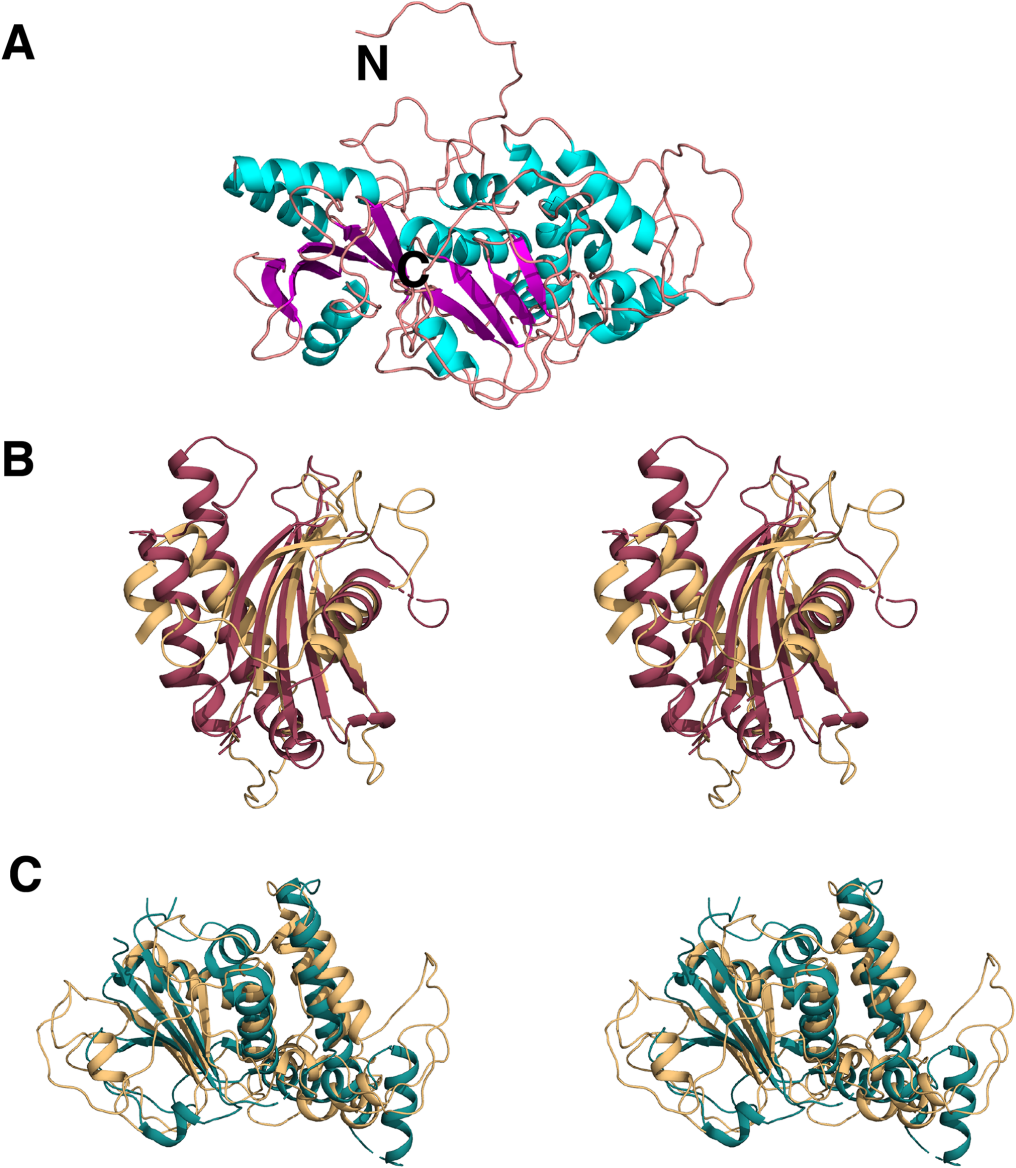
Homology model of C9orf72. (A) Template-based model of C9orf72, generated by RaptorX (Källberg et al., 2012), with α-helices shown in cyan, β-strands in magenta and coil/loop regions in pink. N- and C-termini are labeled. (B) Stereo representation of the superposition of C9orf72 (shown in beige, residues 1–144) with the longin domain of Lst4 (PDB code: 4ZY8; (Pacitto et al., 2015)) shown in raspberry red. (C) Stereo representation of the superposition C9orf72 (shown in beige, residues 195–481) with folliculin (PDB code: 3V42; (Nookala et al., 2012)) shown in deep teal. All figures were rendered using PyMol (www.pymol.org).

SOPMA (Geourjon & Deléage, 1995) and PSIpred (Buchan et al., 2013) predicted the protein to be more alpha-helical in nature. However, no structural hits for the query sequence were found with these programs. RaptorX (Källberg et al., 2012) and LOMETS (Wu & Zhang, 2007), on the other hand, were both successful in generating three-dimensional models of C9orf72 protein from its amino acid sequence. The top template selected by the LOMETS server was 3TW8 with a *Z*-score of ∼0.9. Similarly, the highest ranking homology model generated by RaptorX used the GEF domain of DENND 1B (PDB ID: 3TW8 (Wu et al., 2011)) as a template. This model (Fig. 9A) has a *P*-value of 9.07e-04, and an un-normalized Global Distance Test score of 92, indicating a model of relatively good quality. C9orf72 (Fig. 9A) appears to be mainly an alpha-helical protein. This independent verification of C9orf72 as a DENN-domain containing protein ties in nicely with the findings of Levine et al. (2013).

We also validated the reliability of our model by independent pairwise structure comparison of our model with folliculin (PDB code 3V42; Nookala et al., 2012), the longin domain of Lst4 (PDB code 4ZY8; Pacitto et al., 2015) and also against all the proteins in the PDB library using the servers TM_align (Zhang & Skolnick, 2005) and COFACTOR (Zhang, Freddolino & Zhang, 2017), respectively. TM_align produced scores of 0.67 against folliculin (over 196 residues) and 0.59 against the longin domain of Lst4 (over 144 residues). The longin domain of Lst4 aligns well with the N-terminal end of C9orf72 model (Fig. 9B) whilst folliculin aligns at the C-terminal end of C9orf72 model (Fig. 9C).

In order to shed some light on how C9orf72 is able to recognize and activate different Rab proteins at a structural level, we generated in silico complexes of C9orf72- with Rab proteins 5, 7 and 11, respectively. Since both template-based modeling of C9orf72 using DENND 1B coordinates and pairwise structure comparison with known partial structural homologues of C9orf72 gave reliably similar secondary structure predictions, we used the complex of DENND 1B with Rab35 as the template to model our C9orf72-Rab complexes. Multiple Rab-GTPases have been proposed as potential C9orf72 targets. Rab proteins 5, 7 and 11 were chosen based on the experiments by Farg et al. (2014), where it was shown that these proteins co-immunoprecipitated with C9orf72. Moreover, these three Rab proteins represent not only each of the major subgroups of the Rab-family of small GTPases, they also represent the various stages of the endocytic pathway where these Rabs have been proposed to function.

The homology models of the complexes were all put through validation studies. PROCHECK assessed more than 98% of all the models to be in the allowed region of the Ramachandran plot (Ramachandran, Ramakrishnan & Sasisekharan, 1963). Both ProSA and VERIFY_3D servers predicted the complexes of C9orf72 to be good quality models (Table 2).

**Table 2:** Validation analyses of the C9orf72 homology models.

	C9orf72•Rab5A	C9orf72•Rab7A	C9orf72•Rab11A
ProSA (z-score)	−4.44 (C9orf72) −5.81 (Rab5A)	−4.49 (C9orf72) −5.74 (Rab7A)	−4.64 (C9orf72) −6.23 (Rab11A)
PROCHECK	98.2% (629 aa)	98.0% (641 aa)	98.4% (635 aa)
VERIFY_3D	94.01%	71.19%	78.57%

**Figure 10:**
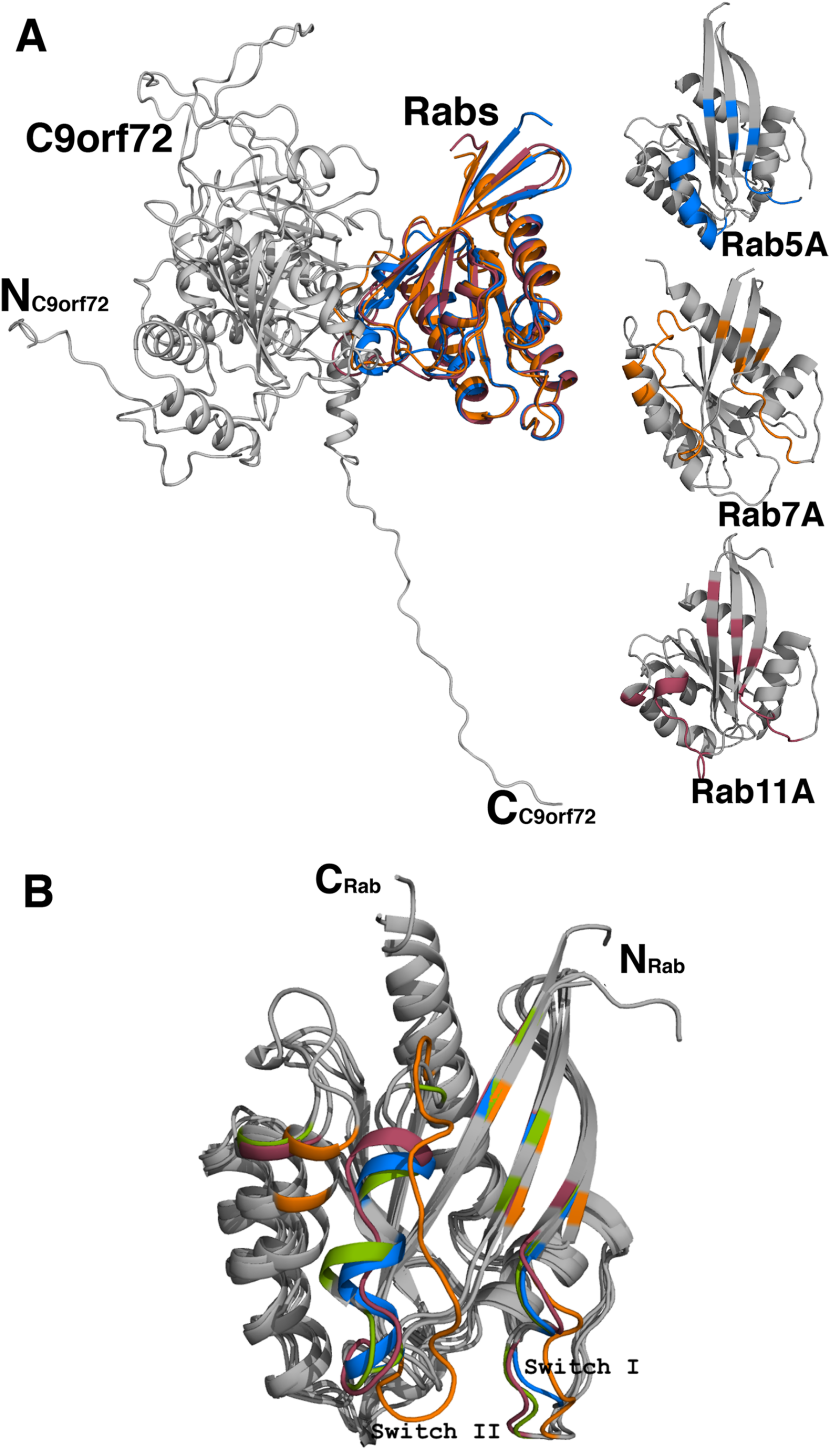
Homology model of C9orf72 and Rab GTPase complexes. (A) Cartoon representation of the superposed complexes of C9orf72 (shown in gray) with the three Rab proteins: Rab5A (marine blue), Rab7A (orange) and Rab11A (raspberry red). The figure also shows the individual Rabs. The main frame of the Rab proteins is colored gray. Residues that interact with C9orf72 at the binding interface are colored accordingly to the convention stated above. (B) Superposition of the Rab proteins from their modeled complexes with C9orf72. The N- and C-termini of the proteins are shown. Also, highlighted are the two canonical loop regions, Switch I and Switch II. All figures were rendered using PyMol (www.pymol.org).

Analyses of the interfaces in the modeled complexes revealed that, as expected, the complexes are very similar in their overall architecture (Fig. 10A). The residues contributed to the interface from the Rab proteins more or less come from the same part of the molecule. The number of residues contributed to the interface is also largely the same in all the three complexes (Table 3). The C9orf72-Rab7A interface seems to have 8–10 more amino acids. This could be because the structure of Rab7A used in the model is longer in length than the other two Rab proteins. A closer look at the binding interface reveals that the only two C9orf72 residues at the binding interface that make hydrogen-bonding interactions with all the four Rab proteins are Arg260 and Ser276 (Table 3). Conformational differences between the three complexes lie mainly in the loop regions of the Rab proteins (Fig. 10B). By analogy to other known Rab structures, these loop regions correspond to biologically important switch regions I and II. These switch regions, located on the surface of the molecules, are essential for the interaction of the Rab proteins with their GEF molecules and participate in the binding interface between the two proteins. Important differences at the molecular level are observed in these switch regions of the three Rab proteins in the complexes. Both, Switch I and Switch II, would have to undergo some degree of conformational change in order to facilitate binding of C9orf72. The putative Rab residues that are involved in interacting with the residues of C9orf72 are listed in Table 3. Additional factors such as association of the Rab proteins with GDP would also influence the conformational change required in these switch regions to facilitate binding of the Rab proteins to C9orf72.

**Table 3:** Potential hydrogen-bonding interactions at the binding interface of the modeled complexes.

C9orf72 residues	Rab5A residues	Rab7A residues	Rab11A residues
Glu89	Ser51 N	Tyr37 OH	Ser4O O
			Ser42 Oγ
Lys90	–	–	–
Gly91	–	Arg69 O	Arg74 Nη1
Glu259	–	Gln71 O	Tyr73 O
Arg260	Arg81 O	Leu73 O	Als75 O
	Ser84 Oγ	Val75 N	
Tyr274	Glu72 Oε1	–	–
Ser276	Ser84 O	Tyr78 OH	Lys13 Nζ
	Met88 O		Ala79 O
			Tyr80 O
Tyr303	–	Gly80 O	–
Thr440	–	–	His112 Nδ1
Glu442	Ser84 O	Val75 O	–
		Gln109 (Oε1 & Nε2)	
Asn446	His83 Nδ1	–	–

**Note:**

Intermolecular contacts between the C9orf72 and Rab proteins were calculated using the program CONTACT (Winn et al., 2011).

## Discussion

We have established a high-yield purification protocol for C9orf72 and three Rab proteins. Furthermore, we have experimentally proven for the first time that C9orf72 is a GEF for three Rab proteins: Rab5A, Rab7A and Rab11A. We also report for the first time that C9orf72 might function as a dual exchange factor based on its ability to activate Rho-GTPases: Cdc42 and RhoA.

C9orf72, engineered to include various N- or C-terminal tags, was initially cloned in *E. coli* using a T7 polymerase expression system. High-level expression was observed but the product aggregated in inclusion bodies. All our efforts to produce the protein in soluble phase were unsuccessful thereby leading us to the use of eukaryotic expression systems. We elaborated an optimized expression and purification protocol that yielded about 1.0 mg of pure C9orf72 from one l of *Sf9* cell culture. C9orf72 has a propensity to form dimers or higher oligomers as indicated by western blot analysis. The Rab proteins on the other hand were expressed and purified from *E. coli* as GST-fusion proteins. CD spectroscopy on the purified proteins showed they were properly refolded.

Far-western analysis performed with C9orf72 gave the first experimental evidence that C9orf72 is able to bind not only all the three Rab proteins but also the two Rho-GTPases we were working with. Size-exclusion chromatography further confirmed these results when we were able to elute the complexes of C9orf72 with all the three Rab proteins, in a buffer containing EDTA. The metal chelator was necessary to disrupt the coordination of Mg^2+^ ion with the Rab proteins and facilitate formation of Rab-GEF complexes. The stoichiometry of the interaction as indicated by SDS–PAGE suggests that C9orf72 binds to Rab5A and Rab11A in 1:1 ratio. The result with Rab7A, on the other hand, suggests that interaction of this GTPase is either unable to dissociate the C9orf72 dimer or shifts the equilibrium toward more C9orf72 dimers, thereby resulting in the formation of 2:1 or 2:2 complexes. The differences in the ratio suggest that perhaps depending on the GTPase involved in the membrane-trafficking pathway, the equilibrium may shift more in favor of C9orf72 dimerization and hence dimeric complexes. Full-length forms of several exchange factors have been shown to function as dimers. It is possible that the same is true in case of C9orf72 as well. This is certainly possible via the N-terminal longin domain of C9orf72. Longin domains are recognized as protein–protein interaction modules that dimerize to provide a platform for GTPase binding.

The DENN-domain is commonly observed in proteins that regulate the membrane trafficking machinery. C9orf72 is thought to function as a GDP/GTP exchange factor for Rab-GTPases, thereby regulating the recruitment of different Rab proteins to specific subcellular membranes, where in turn they can sequester other effector proteins and allow specific intracellular protein localization to take place. The molecular functions, biological processes and the cellular location of C9orf72 predicted by COFACTOR (Zhang, Freddolino & Zhang, 2017) server is in keeping with the insights gained about the protein since its discovery, with Rab-GTPase binding ranked as the top molecular function for C9orf72. Our experiments provide the much-needed evidence that C9orf72 is indeed able to bind Rab-GTPases as a GEF to activate them. The novel finding of C9orf72 acting as a GEF for Rho-GTPases points to a functional interplay between Rab- and Rho-GTPases. Rab-family members regulate vesicular trafficking pathways whilst members of the Rho-GTPase family are pivotal in regulating the cytoskeleton. Crosstalk between these two types of GTPases is rather rare since the action of these molecules is considered to be hierarchical and complementary. Recently, however, it was shown that Rab11 (key regulator in endosomal recycling pathway) and Rac1 (a central player in cytoskeleton regulation) do indeed network on the endosomes via FIP3, a Rab11 effector (Bouchet et al., 2016). So, it is not unreasonable to assume that C9orf72 might act as a GEF for both these GTPase families to facilitate particular cellular functions as yet unknown.

Several membrane sources, including the endoplasmic reticulum, contribute to the autophagosome biogenesis through the participation of specific Rab-GTPases like Rab5, Rab7 and Rab11 amongst other proteins at various stages of autophagy. It is believed that interaction of C9orf72 with different Rab proteins allows it to regulate both membrane trafficking and autophagy (Nassif, Woehlbier & Manque, 2017). Cdc42, a Rho-GTPase member, was recently identified as a regulator of autophagy-related signaling (Till et al., 2015), again suggesting a possible interplay between Rab- and Rho-GTPases could shape context-specific regulation of autophagy and vesicular trafficking. Further studies will be required to unravel this complex networking between the members of two families of GTPases via C9orf72.

All the crystallographic structures of different Rab proteins, elucidated till date, in their GDP- and GTP-bound states show that these proteins adopt two different conformations with most of the GDP/GTP-induced differences occurring in the two switch regions. In keeping with the known DENN-Rab complex, the C-terminal end of C9orf72 makes most contact with the Rab proteins. Analysis of regions predicted to interact with a Rab-GTPase from the homology models revealed that C9orf72 has more polar residues in the binding region. Although the model used is only a predicted structure for C9orf72, the differences in the residues from the three Rab proteins that promote interaction with C9orf72 at the binding interface suggest that C9orf72 uses different combinations of the same blueprint to promote nucleotide exchange. We believe that differences in the conformation of the binding site will allow for the remodeling of the two switch regions in the Rab-GTPases, potentially regulating GEF activity of C9orf72.

The biological relevance of dimerization or the ability of C9orf72 to interact with members of two different GTPase subfamily is yet to be answered. But given that C9orf72 forms dimers and has shown promiscuity by forming complexes with both Rab- and Rho-GTPases to activate them, it is tempting to suggest that C9orf72 might allow for a more complex combinatorial regulation of different intracellular membrane-trafficking events.

## Conclusions

In summary, here we describe a detailed study on the expression, purification and biochemical characterization of C9orf72, an ALS associated protein. Using experimental data we show that C9orf72 is a GEF. In addition, the distinctive presence of both Rab- and Rho-GTPase GEF activities suggests that C9orf72 may function as a dual exchange factor coupling physiological functions such as cytoskeleton modulation and autophagy with endocytosis.

## Supplemental Information

10.7717/peerj.5815/supp-1 Supplemental Information 1 Raw numeric data for plots presented in the manuscript. Raw numeric data for Fig. 8 presented in the manuscript.

## Additional Information and Declarations

### Data Availability

The following information was supplied regarding data availability: The raw data are available as a Supplemental File.
